# Long-term exposure to low-level ambient BTEX and site-specific cancer risk: A national cohort study in the UK Biobank

**DOI:** 10.1016/j.eehl.2025.100146

**Published:** 2025-04-09

**Authors:** Kexin Yu, Ying Xiong, Renjie Chen, Jing Cai, Yaoxian Huang, Haidong Kan

**Affiliations:** a Shanghai Institute of Infectious Disease and Biosecurity, School of Public Health, Key Lab of Public Health Safety of the Ministry of Education and NHC Key Lab of Health Technology Assessment, Fudan University, Shanghai 200032, China; b Department of Civil and Environmental Engineering, Wayne State University, Detroit, MI 48202, USA; c Department of Climate and Space Sciences and Engineering, University of Michigan, Ann Arbor, MI 48109, USA; d Children's Hospital of Fudan University, National Center for Children's Health, Shanghai 201102, China

**Keywords:** Cancer, Benzene, Toluene, Xylene, Cohort study

## Abstract

Benzene, toluene, ethylbenzene, and xylene (BTEX) have been associated with certain cancers in the occupational population. This study aimed to investigate the associations between low-level ambient BTEX exposure and cancer risks in the general population. We leveraged data from the UK Biobank and included individuals free of cancer at 2006–2010 baseline. Annual concentrations of BTEX were estimated using a chemistry-climate model, and the associations between BTEX and incident overall and 18 site-specific cancers were investigated with Cox proportional hazard models. We also fitted restricted cubic splines to explore the exposure-response relationships. The study sample comprised 409,579 participants [mean age 56.2 (8.11) years; 219,315 (53.5%) females]. Over a mean (SD) follow-up period of 11.2 (2.64) years (4,597,164 person-years), 60,777 overall incident cancer cases occurred. The results showed significant associations between overall cancers and benzene [HR 1.93 (95% CI: 1.89, 1.96)], toluene [1.25 (1.23, 1.26)] and xylene [1.11 (1.10, 1.12)]. Benzene and toluene were associated with a higher risk of 18 site-specific cancers. For xylenes (a summation of ethylbenzene, m/p-xylene, and o-xylene in the model), significant associations with multiple myeloma, hepatobiliary tract, thyroid, or connective soft tissue were not observed. Exposure-response curves suggested a higher risk of overall cancer beyond the benzene threshold. For toluene and xylene, there was no threshold or plateau across the range of exposures. This large-scale prospective cohort study demonstrates that long-term exposure to low-level ambient BTEX could increase the risk of overall and site-specific cancers in the general population.

## Highlights

• The first national study to assess the link between low-level ambient BTEX exposure and risk of overall and site-specific cancers.
• Significant links found between BTEX and incidences of overall and site-specific cancers.
• The exposure-response relationship in the general population suggested a threshold effect for benzene.

## Graphical abstract

**Figure:**
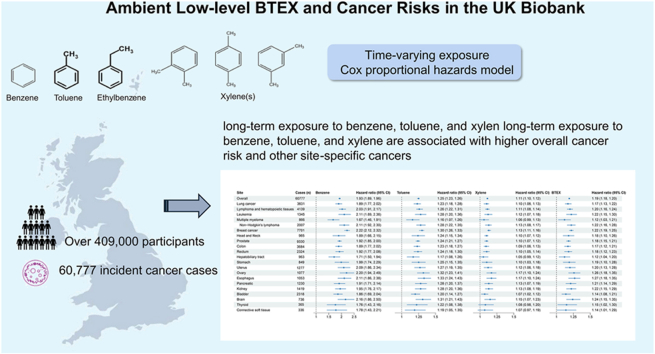

## 1 Introduction

Benzene, toluene, ethylbenzene, and xylene (BTEX) are the most abundant toxic volatile organic compounds (VOCs) found in the outdoor environment [1]. The primary sources of ambient BTEX include combustion of fossil fuel derivatives (i.e., petrol or gasoline) from motor vehicles, aircraft, and industrial emissions [[1], [2], [3], [4]]. As hazardous air pollutants, the carcinogenic effect of BTEX has been examined in experimental, animal, and epidemiologic studies [[5], [6], [7], [8]]. The International Agency for Research on Cancer (IARC) has categorized benzene as a carcinogen to humans. A well-documented causal link exists between benzene and leukemia as well as other hematological cancers, along with indications of a higher lung cancer risk. However, its role in other cancers is not well established. Toluene and xylene are both classified as possible carcinogens. Elevated risks of non-Hodgkin's lymphoma (NHL), Hodgkin's disease, acute myeloid leukemia, and liver and biliary tract cancers have been demonstrated following exposure to organic solvents including toluene or xylene [9]. Occupational studies also pointed to a possible relationship between toluene, xylene and lung, thyroid, prostate, and other general cancers [[10], [11], [12], [13]].

Inhalation of airborne emissions and cigarette smoke are the main sources of BTEX exposure for the general population [14]. Given their widespread presence as environmental contaminants, ambient exposure to BTEX might lead to adverse health effects in the general population. While occupational BTEX exposure has been associated with certain cancer incidence, it remains unclear whether ambient low-level BTEX exposure can affect the risk of cancer among the general population. Existing evidence regarding the carcinogenic potential of BTEX is inconclusive and limited for several reasons: (1) assessment of specific exposure cannot be made in occupational studies since in most circumstances workers are simultaneously exposed to mixture of organic solvents; (2) exposure-response relationship cannot be fitted, as occupational studies often used binary or categorical (i.e., qualitative approaches to classify exposure status as exposed or unexposed) indicator for exposure based on job titles; (3) individual exposure assessment is not available due to lack of monitoring data; (4) occupational studies are often based on high exposure levels and a small number of observed cases; (5) case–control or retrospective study design hinders causal inference.

To assess the association of long-term low-level BTEX exposure with overall as well as site-specific cancer risk among the general population, we conducted a prospective national study with the UK Biobank. We also aimed to explore the exposure-response relationship, providing scientific ground for environmental regulation.

## 2 Methods

### 2.1 Study population

The UK Biobank is a well-established population-based cohort study involving over 500,000 participants aged 40–70 years, enrolled between 2006 and 2010, and followed up through linkage to health administrative datasets [15]. Participants attended assessment centers in the UK and responded to questionnaires on sociodemographic characteristics, lifestyle, and medical information. Of 502,370 participants (43 withdrawals excluded), we excluded participants with cancer prior to baseline assessment based on self-reported records and health administrative dataset (n ​= ​50,734), and those with missing data on covariates or exposures (n ​= ​42,057), resulting in a final sample of 409,579 participants.

### 2.2 Exposure assessment

Annual mean levels of benzene, toluene, xylenes from 2006 to 2021 were derived from a three-dimensional chemistry-climate model, the Community Earth System Model (CESM) Community Atmosphere Model with Chemistry (CAM6-Chem, version 6), which has been widely used to simulate atmospheric composition [[16], [17], [18]]. It is worth noting that xylenes exposure is a summation of ethylbenzene, m/p-xylene, and o-xylene. These species are collectively represented as a single entity in the CESM model, reflecting their combined representation in the input emission inventory. The horizontal resolution of the CESM model is 0.9° latitude by 1.25° longitude. Specifically, the global anthropogenic emission inventory applied in CAM6-Chem model was derived primarily from the Copernicus Atmosphere Monitoring Service (CAMS) version 5.1 [19], which provides global monthly emissions of non-methane volatile organic compounds (NMVOCs) at the horizontal resolution of 0.1° latitude by 0.1° longitude. We compared modeled BTEX concentrations with on-site measurements from European countries, and the comparison showed a normalized mean bias of ±25%. Detailed information regarding the model's performance was previously reported [18]. We assigned the annual average benzene, toluene, xylene, and the average of BTEX concentrations to each participant's residential address record at a resolution of 1 ​km ​× ​1 ​km during the study period.

### 2.3 Cancer incidence ascertainment

We retrieved records on cancer registry (field ID 40006), death certificate (field ID 40001), and hospital admission (field ID 41270) provided by the National Health Service (NHS) Digital (England and Wales) and the NHS Central Register (Scotland). The endpoint for each participant was the first diagnosis of any cancer, and major cancer sites included: lung, lymphoma and hematopoietic tissues, breast, head and neck, prostate, colon, rectum, hepatobiliary tract, stomach, uterus, ovary, esophagus, pancreas, kidney, bladder, brain, thyroid, and connective soft tissue. The cancer sites were chosen based on a prior study from UK Biobank and covered all major cancer sites [20]. We also looked into three subtypes of hematopoietic malignancy: leukemia, multiple myeloma and NHL. All data are coded based on the WHO's International Classification of Diseases, 10th revision (ICD-10). Detailed codes for identifying each cancer were provided in Table S1. For overall cancer, participants were followed until the first date of any cancer diagnosis, death, or end-of-study (2023-05-01), whichever came first. For site-specific analysis, censoring occurred at the diagnosis of the corresponding cancer, death, or the end of follow-up.

### 2.4 Covariates

We selected confounders based on prior knowledge and data availability [21,22]. Data on demographic, socioeconomic, lifestyle and health-related information were collected at baseline assessment. Finally, we included age, gender, ethnicity, educational qualification, household income, Townsend deprivation index (TDI), body mass index (BMI), drinking status, smoking status, physical activity, passive smoking exposure (categorized as “none” or “any”, with “any” referring to secondhand tobacco smoke exposure at home exceeding 1 ​h per week), solid-fuel cooking or heating, annual average concentrations of particular matter with aerodynamic diameter ≤ 2.5 ​μm (PM_2.5_) and nitrogen dioxides (NO_2_) at each participant's address in 2010. TDI is an indicator of neighborhood-level socioeconomic status, where higher positive values represent areas with greater socioeconomic disadvantages. For categorical variables, we used the missing indicator method (i.e., in cases where covariate information was missing, we coded the covariate as “missing” category) [23].

### 2.5 Statistical analysis

Descriptive statistics were used to summarize the baseline characteristics and environmental exposures of the study population. Pearson correlation coefficients between air pollutants were computed. Then, Cox proportional hazards models were applied with time-varying exposure data, incorporating spatiotemporal heterogeneity of BTEX and cancer risk. We adjusted for age, sex, gender, ethnicity, educational qualification, household income, TDI, BMI, drinking status, smoking status, physical activity, passive smoking exposure, solid-fuel usage (cooking or heating), PM_2.5_ and NO_2_ in the main analyses. For breast, uterine, and ovarian cancers, analyses were restricted to females. Similarly, analyses were restricted to male participants for prostate cancer. We evaluated the proportional hazards assumption using Schoenfeld residuals and found no evidence of violation. Regression results were reported as estimated hazard ratios (HRs) associated with each interquartile range (IQR) increment of BTEX concentration with 95% confidence intervals (CIs). The Benjamini-Hochberg procedure was used to control the False Discovery Rate (FDR) at a 5% threshold to address false positives due to multiple testing, and comparisons with FDR-adjusted *P*-values < 0.05 were interpreted as significant [24].

To further explore the exposure-response relationship of BTEX and overall cancer incidence, we used restricted cubic spline with 5 knots distributed across the 5th, 27.5th, 50th, 72.5th, and 95th percentiles of benzene, toluene, and xylenes. We also performed sub-group analyses based on age, sex, BMI, smoking status, and solid-fuel usage to identify susceptible subpopulations.

For sensitivity analyses, we first excluded cancer cases diagnosed within the initial 2 years of follow-up. Then, we also restricted analyses to participants who did not relocate during the study period. We also ran minimum adjusted models that only included age and gender and reported unadjusted results. Statistical analyses were conducted with R software (version 4.1.2). Statistical significance was defined as a two-tailed *P*-value < 0.05.

## 3 Results

Baseline characteristics of included participants are presented in Table 1 by cancer incidence. Our study sample consisted of 409,579 participants [219,315 (53.5%) females] whose mean age at baseline was 56.2 (8.1) years. Of 409,579 participants free of cancer at baseline, 93.7% were white, 67.1% were overweight or obese. Current drinker accounted for 91.9% of the study population. We observed 60,777 cancer incident cases during a mean follow-up of 11.2 years, contributing to 4,597,164 person-years. Compared to non-cases, incident cancer cases during follow-up tended to be older, overweight or obese, and from households with lower income, with lower education levels. Table S2 presents the correlation among air pollutants. There was no correlation between BTEX and PM_2.5_ or NO_2,_ and a moderate correlation between benzene, toluene, and xylene.

**Table 1:** Baseline characteristics of participants included in the study.

Characteristics	All	Any incident cancer	Without incident cancer
Sample size, n	409,579	60,777	348,802
Gender, female, n (%)	219,315 (53.5)	28,454 (46.8)	190,861 (54.7)
Age at baseline, mean (SD), years	56.2 (8.11)	60.0 (6.94)	55.5 (8.12)
Ethnicity, n (%)			
White	383,694 (93.7)	58,790 (96.7)	324,904 (93.1)
Non-white	23,859 (5.8)	1721 (2.8)	22,138 (6.3)
Missing	2026 (0.5)	266 (0.4)	1760 (0.5)
BMI, n (%)			
Normal	134,794 (32.9)	18,627 (30.6)	116,167 (33.3)
Overweight	174,332 (42.6)	26,875 (44.2)	147,457 (42.3)
Obese	100,453 (24.5)	15,275 (25.1)	85,178 (24.4)
Smoking status, n (%)			
Never	225,104 (55.0)	29,905 (49.2)	195,199 (56.0)
Previous	139,830 (34.1)	23,688 (39.0)	116,142 (33.3)
Current	42,561 (10.4)	6863 (11.3)	35,698 (10.2)
Missing	2084 (0.5)	321 (0.5)	1763 (0.5)
Alcohol status, n (%)			
Never	18,258 (4.5)	2723 (4.5)	15,535 (4.5)
Previous	14,763 (3.6)	2196 (3.6)	12,567 (3.6)
Current	376,558 (91.9)	55,858 (91.9)	320,700 (91.9)
Physical activity, n (%)			
Low	62,028 (15.1)	9254 (15.2)	52,774 (15.1)
Moderate	133,004 (32.5)	19,711 (32.4)	113,293 (32.5)
High	133,480 (32.6)	19,557 (32.2)	113,923 (32.7)
Missing	81,067 (19.8)	12,255 (20.2)	68,812 (19.7)
Household income, n (%)			
<£18,000	77,277 (18.9)	13,394 (22.0)	63,883 (18.3)
£18,000–£30,999	87,908 (21.5)	14,337 (23.6)	73,571 (21.1)
£31,000–£51,999	91,199 (22.3)	12,383 (20.4)	78,816 (22.6)
≥£52,000	71,764 (17.5)	8499 (14.0)	63,265 (18.1)
Missing	81,431 (19.9)	12,164 (20.0)	69,267 (19.9)
Townsend deprivation index	−1.3 (3.05)	−1.52 (2.98)	−1.3 (3.06)
Education qualification, n (%)			
None	67,263 (16.4)	12,823 (21.1)	54,440 (15.6)
O levels, GCSEs, CSEs or equivalent	69,845 (17.1)	9859 (16.2)	59,986 (17.2)
A levels/AS levels or equivalent	21,992 (5.4)	3025 (5.0)	18,967 (5.4)
NVQ, HND, HNC or other professional	112,434 (27.5)	16,237 (26.7)	96,197 (27.6)
College or University degree	129,785 (31.7)	17,440 (28.7)	112,345 (32.2)
Missing	8260 (2.0)	1393 (2.3)	6867 (2.0)
Solid-fuel cooking or heating, n (%)			
None	394,059 (96.2)	58,192 (95.7)	335,867 (96.3)
Yes	10,615 (2.6)	1725 (2.8)	8890 (2.5)
Missing	4905 (1.2)	860 (1.4)	4045 (1.2)
Passive smoking exposure, n (%)			
None	348,247 (85.0)	51,143 (84.1)	297,104 (85.2)
Any	20,515 (5.0)	2986 (4.9)	17,529 (5.0)
Missing	40,817 (10.0)	6648 (10.9)	34,169 (9.8)
PM_2.5_ (μg/m^3^)	10.0 (1.06)	9.95 (1.05)	10.0 (1.06)
NO_2_ (μg/m^3^)	26.7 (7.63)	26.2 (7.49)	26.8 (7.65)

BMI, body mass index; SD, standard error; GCSE, General Certificate of ​Secondary Education; CSE, Certificate of Secondary Education; NVQ, National Vocational Qualification; HND, Higher National Diploma; HNC, Higher National Certificate; ​PM_2.5_, annual average level of particular matter with aerodynamic diameter < 2.5 ​μm; NO_2_, annual average level of nitrogen dioxides.

**Fig. 1:**
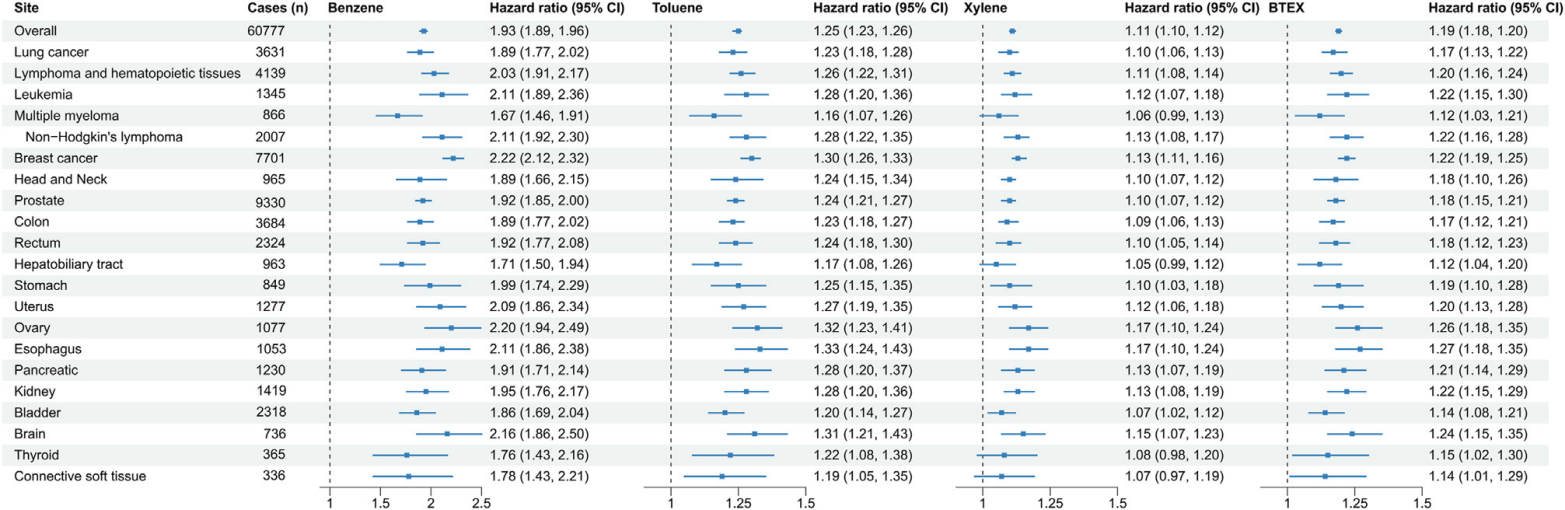
Associations of long-term exposure to benzene, toluene, and xylene with risk of overall and site-specific cancer. Models were adjusted for age, sex, ethnicity, body mass index, drinking status, smoking status, physical activity, educational qualification, household income, Townsend deprivation index, passive smoking exposure, solid-fuel usage, PM_2.5_ and NO_2._ Associations were presented as Hazard Ratios (95% CIs) per interquartile range increases in concentrations of benzene (0.05 ​ppb), toluene (0.03 ​ppb), xylene (0.15 ​ppb) and BTEX (0.22 ​ppb). CI, confidence interval.

Long-term BTEX exposure was associated with elevated incident cancer risk after adjusting for multiple comparisons (Fig. 1, Table S3). Each IQR increase in benzene, toluene, and xylene concentrations corresponded to a higher overall cancer risk [HR 1.93 (95% CI: 1.89, 1.96), 1.25 (1.23, 1.26), and 1.11 (1.10, 1.12)]. The risk estimates for site-specific cancers per IQR increase in benzene were highest for leukemia [HR 2.11 (95% CI: 1.89, 2.36)], NHL [2.11 (1.92, 2.30)], breast cancer [2.22 (2.12, 2.32)], ovary cancer [2.20 (1.94, 2.49)], and brain cancer [2.16 (1.86, 2.50)]. Similar associations were found for toluene but smaller in terms of effect size. We did not observe significant associations of multiple myeloma, hepatobiliary tract cancer, thyroid cancer, or connective soft tissue cancer with xylene. For subtypes of hematopoietic malignancy, estimated HRs (95% CIs) were 2.11 (1.89, 2.36) for leukemia, 2.11 (1.92, 2.36) for NHL and 1.67 (1.46, 1.91) for multiple myeloma corresponding to each IQR increase in benzene. Similar associations were observed for toluene and xylene. The FDR-adjusted *P*-values for the association between BTEX and cancer risks are presented in Table S3.

**Fig. 2:**
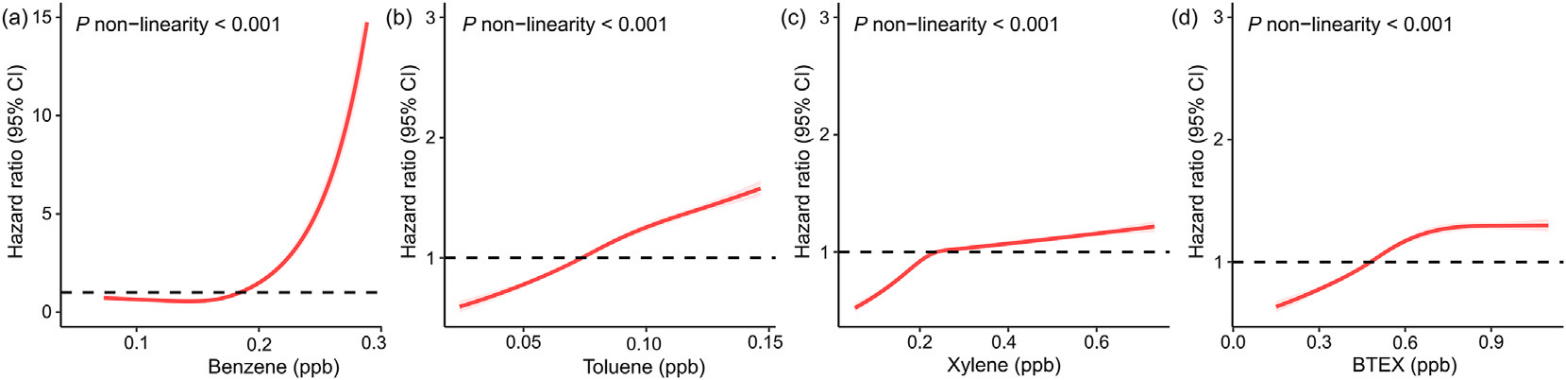
Exposure-response curves for the associations of benzene, toluene, and xylene with overall cancer incidence. Models were adjusted for age, sex, ethnicity, body mass index, drinking status, smoking status, physical activity, educational qualification, household income, Townsend deprivation index, passive smoking exposure, solid-fuel usage, PM_2.5_ and NO_2._

The exposure-response curve demonstrates nonlinear increase in overall cancer risk as benzene concentrations increase (Fig. 2) (*P* for non-linearity <0.001). The association between long-term benzene exposure and overall cancer was nonsignificant below a threshold of 0.18 ​ppb, then increased drastically beyond the threshold point. For toluene and xylene, there was a monotonic increase in the exposure-response association, with a steeper slope for toluene within the observed exposure range. Exposure-response curves for subtypes of hematologic malignancy were presented in Figs. S1–S3. In sub-group analyses, younger individuals, non-white participants, and females were generally more sensitive towards the hazardous effect of BTEX (Figs. S4–S9).

The results remained consistent in sensitivity analyses excluding cancer cases occurring within the initial 2 years or restricting the analyses to participants who did not relocate during the follow-up (Figs. S10–S11). The associations of long-term benzene, toluene, and xylene exposure with overall cancer risk remained significant. Furthermore, in the unadjusted model, the results did not alter significantly (Table S4).

## 4 Discussion

In this prospective cohort consisting of 409,579 participants in the UK, we assessed the association of long-term exposure to ambient low-level BTEX with overall cancer as well as 18 site-specific cancers. To date, this is the first cohort study to assess the risks of overall and site-specific cancers associated with individual time-varying exposure to ambient low-level BTEX in the general population. We found that long-term exposure to BTEX was associated with higher overall and site-specific cancer risk. Furthermore, there was some discrepancy in the association of different subtypes of hematopoietic malignancy (i.e., leukemia, multiple myeloma, and NHL). Exposure-response curves suggested a monotonic increase in overall cancer risk associated with higher levels of BTEX, with the highest effect estimates for benzene, followed by toluene and xylenes.

We found a significant increase in overall and 18 site-specific cancer risks linked to long-term benzene exposure. The effects of benzene were strongest for leukemia, NHL, and cancers of breast, ovary, uterus, brain, and esophagus. The association between occupational benzene exposure and leukemia is well-established based on evidence in the general population [25]. A cohort study across 23 centers in 10 European countries found a non-significant increased hazard ratio of leukemia for high-exposure group classified based on self-reported occupations and job exposure matrix [26]. For NHL and multiple myeloma, the primary outcome was mortality, which limited the ascertainment of cancer incidence [27,28]. A study among 73,789 workers exposed to benzene and followed up over 28 years reported a positive association between benzene, leukemia and NHL [29]. A population-based cohort study in Shanghai female workers showed a significantly higher NHL risk associated with occupational benzene exposure [30]. Our results also support previous occupational cohort studies on hematopoietic malignancy. Furthermore, we compared the effect estimates of benzene on three subtypes, leukemia, multiple myeloma, and NHL, and found a stronger association for leukemia and NHL. Mechanisms underlying the observed heterogeneity remain to be defined. A recent meta-analysis concluded that there were harmonious and robust associations between occupational benzene exposure and lung cancer [31]. Epidemiologic evidence on benzene exposure and cancer sites other than the ones previously discussed in non-occupational population is rather limited. Prior epidemiological studies of highly exposed populations (i.e., occupational populations) also reported suggestive association between benzene (or solvents that included benzene) and cancers of the stomach [32], cancers of the colon and rectum [29], cancers of the prostate and bladder [33], kidney cancer [34], and cancers of central nervous system [35], as well as the pancreas [36].

Similarly, in the present study, we reported a significant association between long-term ambient toluene exposure and overall cancer and 18 site-specific cancers. Numerous epidemiologic studies have investigated toluene exposure as a potential risk factor for cancer. The discrepancy in results of particular cancer types across studies hinders assessment of the carcinogenic potential of toluene. A study utilized monitoring data to estimate ambient toluene exposure and found no association with overall cancer but identified a link with respiratory tract cancers [37]. A cohort study of over 7000 shoe factory workers reported excess risk for trachea, bronchus and lung cancer associated with exposure to solvents that included toluene [38]. Another study examined cancer incidence among 6830 male workers in printing plants, and found higher connective tissue cancer mortality risk among exposed workers [39]. Recently a study found a positive connection between blood toluene level and thyroid cancer risk [12]. Many other epidemiologic studies showing higher risks of cancer were based on individuals concurrently exposed to multiple chemicals [40]. Future investigations based on individual exposure are warranted to confirm our findings.

For xylene, we did not find a significant association with multiple myeloma, hepatobiliary tract, thyroid, or connective soft tissue cancer. Epidemiologic evidence linking xylene exposure to overall cancer and cancer at specific sites is limited. Two studies suggested a possible association of xylene exposure with leukemia among solvent-exposed workers [32,41]. A retrospective cohort study revealed no multiple myeloma or NHL cases among xylene-exposed workers [42]. Another case–control reported increased risks for colon and rectum cancers following high exposure to xylene [43]. Relatively small size and lack of concentration measurement precluded a conclusive reference on carcinogenic potential of xylene.

The exposure-response curves between long-term BTEX exposure and overall cancer risk in this study showed a consistent increase without a discernible threshold. This is in accordance with a recent study on benzene exposure and cancer mortality showing monotonically increasing relationships [22]. In general, benzene is the only compound among BTEX for which some countries have specific air quality standards with noticeable differences in the limits, while toluene and xylene are generally regulated within the category of VOCs. The UK adopted the EU's approach, setting the annual benzene limit at 5 ​μg/m^3^, whereas the WHO does not define a specific safe level for benzene exposure but provides risk-based estimates [44]. In the case of China, there is no specific ambient air quality standard for benzene. For toluene and xylene, there is currently no specific standard in the UK. The US Environmental Protection Agency tracks ambient benzene concentrations and assesses associated health risks but does not have a national standard for BTEX. The UK Non-Automatic Hydrocarbon Network monitors ambient benzene concentrations, and the Department for Environment, Food & Rural Affairs monitors toluene, ethylbenzene, and xylenes using an advanced automatic PerkinElmer gas chromatograph at urban, industrial and rural locations [45]. In 2015, the annual mean surface BTEX concentrations were up to 30 ​μg/m^3^ for Eastern China, India, Southeast Asia, Europe, Western Canada, and Eastern US [4]. In our study, long-term BTEX exposure, even at levels below the current limit, could potentially increase overall and site-specific cancer risk. Our findings suggest the necessity and urgency of integrating the management of BTEX compounds into air quality and industrial emission control policies.

The mechanisms underlying the carcinogenicity of BTEX are largely unknown. Shared pathways may include increased mitochondrial copy numbers, telomere elongation, compromised DNA damage repair, disruptions in non-coding RNA expression, and epigenetic alterations [[6], [7], [8]]. Another proposed pathway contributing to carcinogenicity potential of BTEX is oxidative stress, leading to gene expression change and DNA damage [46]. *In vivo* and *in vitro* studies suggested that benzene, toluene and xylene can induce hyperphosphorylation of p53, which is involved in tumor promotion [47]. While benzene is an experimental carcinogen, evidence suggests that toluene and xylene exhibit carcinogenic potential at high concentrations in experimental animals [48].

Our study presents notable strengths in terms of exposure assessment and causal evaluation. First, we were able to assess individual time-varying exposure of BTEX. The majority of previous epidemiologic studies based on highly exposed workers assessed BTEX exposure qualitatively, thus incapable of estimating specific risk corresponding to BTEX exposure or establishing exposure-response relationships between BTEX and cancer risk. Furthermore, studies in occupational settings usually involve benzene exposure alongside other solvent exposures. With high-resolution exposure assessment models, we specifically calculated individual BTEX exposure and compared effect estimates of benzene, toluene, and xylene in the same population. Second, by virtue of a large prospective cohort and decent case identification accuracy, as well as substantial adjustment for confounders, we provided robust evidence for causal assessment on BTEX and cancer. Last, we conducted a comprehensive cancer-wide study to examine the associations between BTEX and 18 site-specific cancers. Nevertheless, certain limitations should be addressed. The exposure assessment based on residential address could not capture activity patterns of individuals, thus potential exposure misclassification might exist. Moreover, despite the adjustment of a series of confounders, we could not rule out residual confounding by other unmeasured factors that might affect the exposure and cancer incidence. Finally, indoor emissions are an important source of BTEX. The lack of data on individual indoor exposure is a common limitation in environmental epidemiological research, and the results should be interpreted with caution.

## 5 Conclusion

Our results suggest a link between long-term low-level ambient BTEX exposure and overall cancer incidence and 18 site-specific cancers in the UK general population. Our findings carry significant public health implications and highlight the need for further research to corroborate these associations and contribute to the evidence of carcinogenic potential of BTEX.

## CRediT authorship contribution statement

**Kexin Yu:** Writing – review & editing, Writing – original draft, Visualization, Software, Methodology, Formal analysis, Conceptualization. **Ying Xiong:** Writing – review & editing, Writing – original draft, Software, Methodology, Formal analysis. **Renjie Chen:** Writing – review & editing, Supervision, Resources, Project administration, Conceptualization. **Jing Cai:** Writing – review & editing, Supervision, Methodology, Data curation. **Yaoxian Huang:** Writing – review & editing, Supervision, Project administration, Funding acquisition, Data curation, Conceptualization. **Haidong Kan:** Writing – review & editing, Supervision, Resources, Project administration, Methodology, Funding acquisition, Conceptualization.

## Declaration of competing interests

The authors declare no conflict of interest.
