# Supplementary material for: Long-term exposure to low-level ambient BTEX and site-specific cancer risk: A national cohort study in the UK Biobank
Source: Eco Environ Health. 2025 Apr 9;4(2):100146. doi: 10.1016/j.eehl.2025.100146 (PMC12136791; doi:10.1016/j.eehl.2025.100146)
Supplement: Multimedia component 1 [file mmc1.docx]

**Supplementary information**

**Long-term exposure to low-level ambient BTEX and site-specific cancer risk: A national cohort study in the UK Biobank**

Kexin Yu, Ying Xiong, Renjie Chen, Jing Cai, Yaoxian Huang, Haidong Kan

**Table of contents**

**Supplementary tables**

**Table S1.** ICD-10 codes for site-specific incident cancer identification

**Table S2.** Pearson correlations of long-term BTX exposure

**Table S3**. Associations of long-term exposure to benzene, toluene, and xylene with risk of overall and site-specific cancer

**Table S4**. Unadjusted model for associations of long-term exposure to benzene, toluene, and xylene with risk of overall and site-specific cancer

**Supplementary figures**

**Figure S1.** Exposure-response curves for the associations of benzene, toluene, and xylene with leukemia incidence

**Figure S2.** Exposure-response curves for the associations of benzene, toluene, and xylene with multiple myeloma incidence

**Figure S3.** Exposure-response curves for the associations of benzene, toluene, and xylene with non-Hodgkin’s lymphoma incidence

**Figure S4**. Associations of long-term exposure to benzene, toluene, and xylene with risk of overall and site-specific cancer stratified by age

**Figure S5**. Associations of long-term exposure to benzene, toluene, and xylene with risk of overall and site-specific cancer stratified by sex

**Figure S6**. Associations of long-term exposure to benzene, toluene, and xylene with risk of overall and site-specific cancer stratified by body mass index

**Figure S7**. Associations of long-term exposure to benzene, toluene, and xylene with risk of overall and site-specific cancer stratified by ethnicity

**Figure S8**. Associations of long-term exposure to benzene, toluene, and xylene with risk of overall and site-specific cancer stratified by smoking status

**Figure S9**. Associations of long-term exposure to benzene, toluene, and xylene with risk of overall and site-specific cancer stratified by solid-fuel usage

**Figure S10.** Associations of long-term exposure to benzene, toluene, and xylene with risk of overall and site-specific cancer excluding cancer cases occurring over first 2 years of follow-up

**Figure S11.** Associations of long-term exposure to benzene, toluene, and xylene with risk of overall and site-specific cancer restricting to non-movers during the follow-up period

**Table S1.** ICD-10 codes for site-specific incident cancer identification

| **Cancer site** | **ICD10** |
| --- | --- |
| All cancer | C00-C97 |
| Lung | C33-C34 |
| Lymphoma and hematopoietic tissues | C81-C96 |
| Leukemia | C91-C95 |
| Multiple myeloma | C90 |
| Non-Hodgkin’s lymphoma | C82–C85 |
| Breast | C50 |
| Head and Neck | C00-C14 |
| Prostate | C61 |
| Colon | C18 |
| Rectum | C19-C20 |
| Hepatobiliary tract | C22-C24 |
| Stomach | C16 |
| Uterus | C54 |
| Ovary | C56 |
| Esophagus | C15 |
| Pancreatic | C25 |
| Kidney | C64-C65 |
| Bladder | C66-C67 |
| Brain | C71 |
| Thyroid | C73 |
| Connective soft tissue | C49 |

**Table S2.** Pearson correlations of long-term BTX exposure

|  | Benzene | Toluene | Xylene | BTX | NO_2_ |
| --- | --- | --- | --- | --- | --- |
| Toluene | 0.82 |  |  |  |  |
| Xylene | 0.63 | 0.95 |  |  |  |
| BTX | 0.74 | 0.99 | 0.99 |  |  |
| NO_2_ | <0.01 | <0.01 | <0.01 | <0.01 |  |
| PM_2.5_ | <0.01 | <0.01 | <0.01 | <0.01 | 0.86 |

NO_2_, nitrogen dioxide; PM_2.5_, fine particulate matter.

**Table S3**. Associations of long-term exposure to benzene, toluene, and xylene with risk of overall and site-specific cancer

|  | Benzene | | Toluene | | Xylene | | BTEX | |
| --- | --- | --- | --- | --- | --- | --- | --- | --- |
|  | Hazard Ratio  (95% CI) | Adjusted *P*-value | Hazard Ratio  (95% CI) | Adjusted *P*-value | Hazard Ratio  (95% CI) | Adjusted *P*-value | Hazard Ratio (95% CI) | Adjusted *P*-value |
| Overall | 1.93 (1.89, 1.96) | <0.001 | 1.25 (1.23, 1.26) | <0.001 | 1.11 (1.1, 1.12) | <0.001 | 1.19 (1.18, 1.20) | <0.001 |
| Lung cancer | 1.89 (1.77, 2.02) | <0.001 | 1.23 (1.18, 1.28) | <0.001 | 1.10 (1.06, 1.13) | <0.001 | 1.17 (1.13, 1.22) | <0.001 |
| Lymphoma and hematopoietic tissues | 2.03 (1.91, 2.17) | <0.001 | 1.26 (1.22, 1.31) | <0.001 | 1.11 (1.08, 1.14) | <0.001 | 1.20 (1.16, 1.24) | <0.001 |
| Leukemia | 2.11 (1.89, 2.36) | <0.001 | 1.28 (1.20, 1.36) | <0.001 | 1.12 (1.07, 1.18) | <0.001 | 1.22 (1.15, 1.30) | <0.001 |
| Multiple myeloma | 1.67 (1.46, 1.91) | <0.001 | 1.16 (1.07, 1.26) | <0.001 | 1.06 (0.99, 1.13) | 0.109 | 1.12 (1.03, 1.21) | 0.007 |
| Non-Hodgkin’s lymphoma | 2.11 (1.92, 2.30) | <0.001 | 1.28 (1.22, 1.35) | <0.001 | 1.13 (1.08, 1.17) | <0.001 | 1.22 (1.16, 1.28) | <0.001 |
| Breast cancer | 2.22 (2.12, 2.32) | <0.001 | 1.30 (1.26, 1.33) | <0.001 | 1.13 (1.11, 1.16) | <0.001 | 1.22 (1.19, 1.25) | <0.001 |
| Head and Neck | 1.89 (1.66, 2.15) | <0.001 | 1.24 (1.15, 1.34) | <0.001 | 1.10 (1.07, 1.12) | 0.002 | 1.18 (1.10, 1.26) | <0.001 |
| Prostate | 1.92 (1.85, 2.00) | <0.001 | 1.24 (1.21, 1.27) | <0.001 | 1.10 (1.07, 1.12) | <0.001 | 1.18 (1.15, 1.21) | <0.001 |
| Colon | 1.89 (1.77, 2.02) | <0.001 | 1.23 (1.18, 1.27) | <0.001 | 1.09 (1.06, 1.13) | <0.001 | 1.17 (1.12, 1.21) | <0.001 |
| Rectum | 1.92 (1.77, 2.08) | <0.001 | 1.24 (1.18, 1.30) | <0.001 | 1.10 (1.05, 1.14) | <0.001 | 1.18 (1.12, 1.23) | <0.001 |
| Hepatobiliary tract | 1.71 (1.5,0 1.94) | <0.001 | 1.17 (1.08, 1.26) | <0.001 | 1.05 (0.99, 1.12) | 0.103 | 1.12 (1.04, 1.20) | 0.004 |
| Stomach | 1.99 (1.74, 2.29) | <0.001 | 1.25 (1.15, 1.35) | <0.001 | 1.10 (1.03, 1.18) | 0.003 | 1.19 (1.10, 1.28) | <0.001 |
| Uterus | 2.09 (1.86, 2.34) | <0.001 | 1.27 (1.19, 1.35) | <0.001 | 1.12 (1.06, 1.18) | <0.001 | 1.20 (1.13, 1.28) | <0.001 |
| Ovary | 2.20 (1.94, 2.49) | <0.001 | 1.32 (1.23, 1.41) | <0.001 | 1.17 (1.10, 1.24) | <0.001 | 1.26 (1.18, 1.35) | <0.001 |
| Esophagus | 2.11 (1.86, 2.38) | <0.001 | 1.33 (1.24, 1.43) | <0.001 | 1.17 (1.10, 1.24) | <0.001 | 1.27 (1.18, 1.35) | <0.001 |
| Pancreatic | 1.91 (1.71, 2.14) | <0.001 | 1.28 (1.2,0 1.37) | <0.001 | 1.13 (1.07, 1.19) | <0.001 | 1.21 (1.14, 1.29) | <0.001 |
| Kidney | 1.95 (1.76, 2.17) | <0.001 | 1.28 (1.20, 1.36) | <0.001 | 1.13 (1.08, 1.19) | <0.001 | 1.22 (1.15, 1.29) | <0.001 |
| Bladder | 1.86 (1.69, 2.04) | <0.001 | 1.20 (1.14, 1.27) | <0.001 | 1.07 (1.02, 1.12) | <0.001 | 1.14 (1.08, 1.21) | <0.001 |
| Brain | 2.16 (1.86, 2.50) | <0.001 | 1.31 (1.21, 1.43) | <0.001 | 1.15 (1.07, 1.23) | <0.001 | 1.24 (1.15, 1.35) | <0.001 |
| Thyroid | 1.76 (1.43, 2.16) | <0.001 | 1.22 (1.08, 1.38) | 0.003 | 1.08 (0.98, 1.20) | 0.128 | 1.15 (1.02, 1.30) | 0.024 |
| Connective soft tissue | 1.78 (1.43, 2.21) | <0.001 | 1.19 (1.05, 1.35) | 0.014 | 1.07 (0.97, 1.19) | 0.184 | 1.14 (1.01, 1.29) | 0.046 |

CI, confidence interval. Note: Models were adjusted for age, sex, ethnicity, body mass index, drinking status, smoking status, physical activity, education qualification, household income, Townsend Deprivation Index, passive smoking exposure, particulate matter with an aerodynamic diameter ≤ 2.5 µm (PM_2.5_) and nitrogen dioxide (NO_2_)_._ Associations were presented as Hazard Ratios (95% CIs) per interquartile range increases in concentrations of benzene (0.05 ppb), toluene (0.03 ppb), xylene (0.15 ppb) and BTEX (0.22 ppb).

*P*-values were adjusted for multiple comparison with the Benjamini-Hochberg procedure.

**Table S4**. Unadjusted model for associations of long-term exposure to benzene, toluene, and xylene with risk of overall and site-specific cancer

|  | Benzene | | Toluene | | Xylene | | BTEX | |
| --- | --- | --- | --- | --- | --- | --- | --- | --- |
|  | Hazard Ratio  (95% CI) | Adjusted *p*-value | Hazard Ratio  (95% CI) | Adjusted *p*-value | Hazard Ratio  (95% CI) | Adjusted *p*-value | Hazard Ratio  (95% CI) | Adjusted *p*-value |
| Overall | 1.96 (1.93, 1.99) | <0.01 | 1.25 (1.24, 1.26) | <0.01 | 1.11 (1.10, 1.12) | <0.001 | 1.19 (1.18, 1.20) | <0.001 |
| Lung cancer | 1.96 (1.84, 2.10) | <0.01 | 1.23 (1.19, 1.28) | <0.01 | 1.10 (1.07, 1.14) | <0.001 | 1.18 (1.14, 1.23) | <0.001 |
| Lymphoma and hematopoietic tissues | 2.12 (1.90, 2.37) | <0.01 | 1.28 (1.20, 1.36) | <0.01 | 1.12 (1.07, 1.18) | <0.001 | 1.22 (1.15, 1.29) | <0.001 |
| Leukemia | 2.04 (1.91, 2.17) | <0.01 | 1.26 (1.22, 1.31) | <0.01 | 1.11 (1.08, 1.14) | <0.001 | 1.20 (1.16, 1.24) | <0.001 |
| Multiple myeloma | 1.67 (1.46, 1.91) | <0.01 | 1.16 (1.07, 1.25) | <0.01 | 1.05 (0.99, 1.13) | 0.121 | 1.11 (1.03, 1.20) | 0.009 |
| Non-Hodgkin’s lymphoma | 2.12 (1.94, 2.32) | <0.01 | 1.28 (1.22, 1.35) | <0.01 | 1.12 (1.08, 1.17) | <0.001 | 1.22 (1.16, 1.28) | <0.001 |
| Breast cancer | 2.22 (2.12, 2.33) | <0.01 | 1.29 (1.26, 1.32) | <0.01 | 1.14 (1.11, 1.16) | <0.001 | 1.22 (1.19, 1.25) | <0.001 |
| Head and Neck | 1.91 (1.68, 2.18) | <0.01 | 1.24 (1.15, 1.33) | <0.01 | 1.10 (1.04, 1.17) | 0.002 | 1.18 (1.10, 1.27) | <0.001 |
| Prostate | 1.94 (1.86, 2.02) | <0.01 | 1.24 (1.21, 1.27) | <0.01 | 1.09 (1.07, 1.11) | <0.001 | 1.18 (1.15, 1.20) | <0.001 |
| Colon | 1.91 (1.79, 2.04) | <0.01 | 1.22 (1.18, 1.27) | <0.01 | 1.09 (1.06, 1.13) | <0.001 | 1.17 (1.13, 1.21) | <0.001 |
| Rectum | 1.94 (1.78, 2.10) | <0.01 | 1.23 (1.17, 1.29) | <0.01 | 1.10 (1.05, 1.14) | <0.001 | 1.18 (1.12, 1.23) | <0.001 |
| Hepatobiliary tract | 1.73 (1.52, 1.96) | <0.01 | 1.16 (1.08, 1.25) | <0.01 | 1.05 (0.99, 1.12) | 0.097 | 1.12 (1.04, 1.20) | 0.004 |
| Stomach | 2.02 (1.76, 2.32) | <0.01 | 1.25 (1.15, 1.35) | <0.01 | 1.11 (1.04, 1.18) | 0.003 | 1.19 (1.10, 1.28) | <0.001 |
| Uterus | 2.08 (1.85, 2.33) | <0.01 | 1.26 (1.18, 1.34) | <0.01 | 1.12 (1.06, 1.18) | <0.001 | 1.20 (1.13, 1.28) | <0.001 |
| Ovary | 2.20 (1.94, 2.49) | <0.01 | 1.32 (1.23, 1.41) | <0.01 | 1.17 (1.10, 1.24) | <0.001 | 1.26 (1.18, 1.35) | <0.001 |
| Esophagus | 2.16 (1.91, 2.45) | <0.01 | 1.33 (1.24, 1.43) | <0.01 | 1.17 (1.11, 1.24) | <0.001 | 1.27 (1.19, 1.36) | <0.001 |
| Pancreatic | 1.93 (1.72, 2.16) | <0.01 | 1.28 (1.20, 1.36) | <0.01 | 1.13 (1.07, 1.19) | <0.001 | 1.21 (1.14, 1.29) | <0.001 |
| Kidney | 1.98 (1.78, 2.20) | <0.01 | 1.27 (1.20, 1.35) | <0.01 | 1.14 (1.08, 1.19) | <0.001 | 1.22 (1.15, 1.30) | <0.001 |
| Bladder | 1.90 (1.75, 2.07) | <0.01 | 1.22 (1.16, 1.27) | <0.01 | 1.08 (1.04, 1.13) | <0.001 | 1.16 (1.11, 1.21) | <0.001 |
| Brain | 2.17 (1.87, 2.52) | <0.01 | 1.30 (1.20, 1.42) | <0.01 | 1.15 (1.07, 1.23) | <0.001 | 1.24 (1.15, 1.35) | <0.001 |
| Thyroid | 1.75 (1.42, 2.16) | <0.01 | 1.21 (1.08, 1.37) | <0.01 | 1.08 (0.98, 1.20) | 0.127 | 1.15 (1.02, 1.30) | 0.024 |
| Connective soft tissue | 1.79 (1.44, 2.23) | <0.01 | 1.19 (1.05, 1.35) | <0.01 | 1.07 (0.97, 1.19) | 0.190 | 1.14 (1.01, 1.29) | 0.047 |

Models were adjusted for age and sex.

*P*-values were adjusted for multiple comparison with the Benjamini-Hochberg procedure.

**
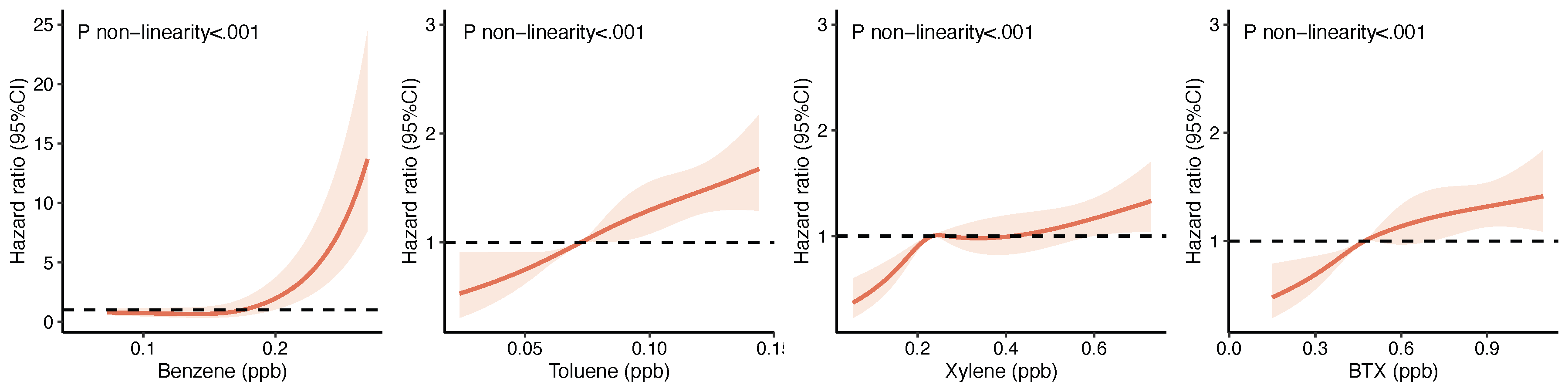
**

**Figure S1.** Exposure-response curves for the associations of benzene, toluene, and xylene with leukemia incidence

**
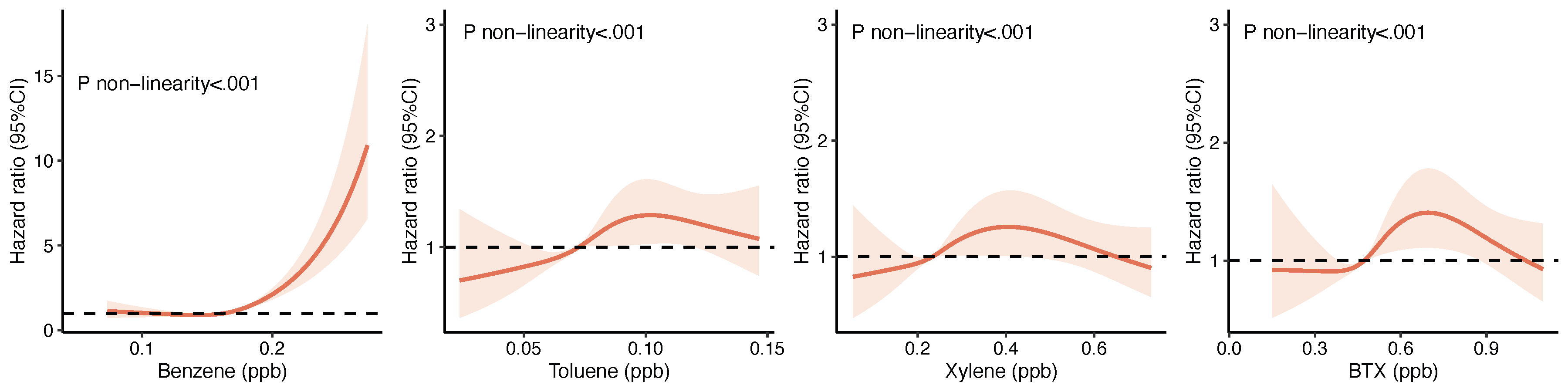
**

**Figure S2.** Exposure-response curves for the associations of benzene, toluene, and xylene with multiple myeloma incidence


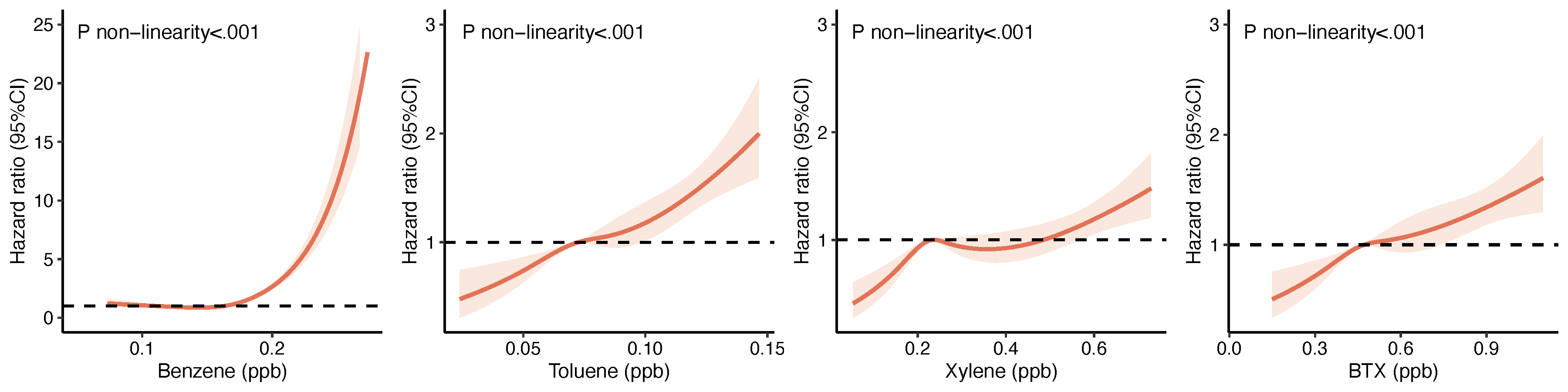


**Figure S3.** Exposure-response curves for the associations of benzene, toluene, and xylene with non-Hodgkin’s lymphoma incidence


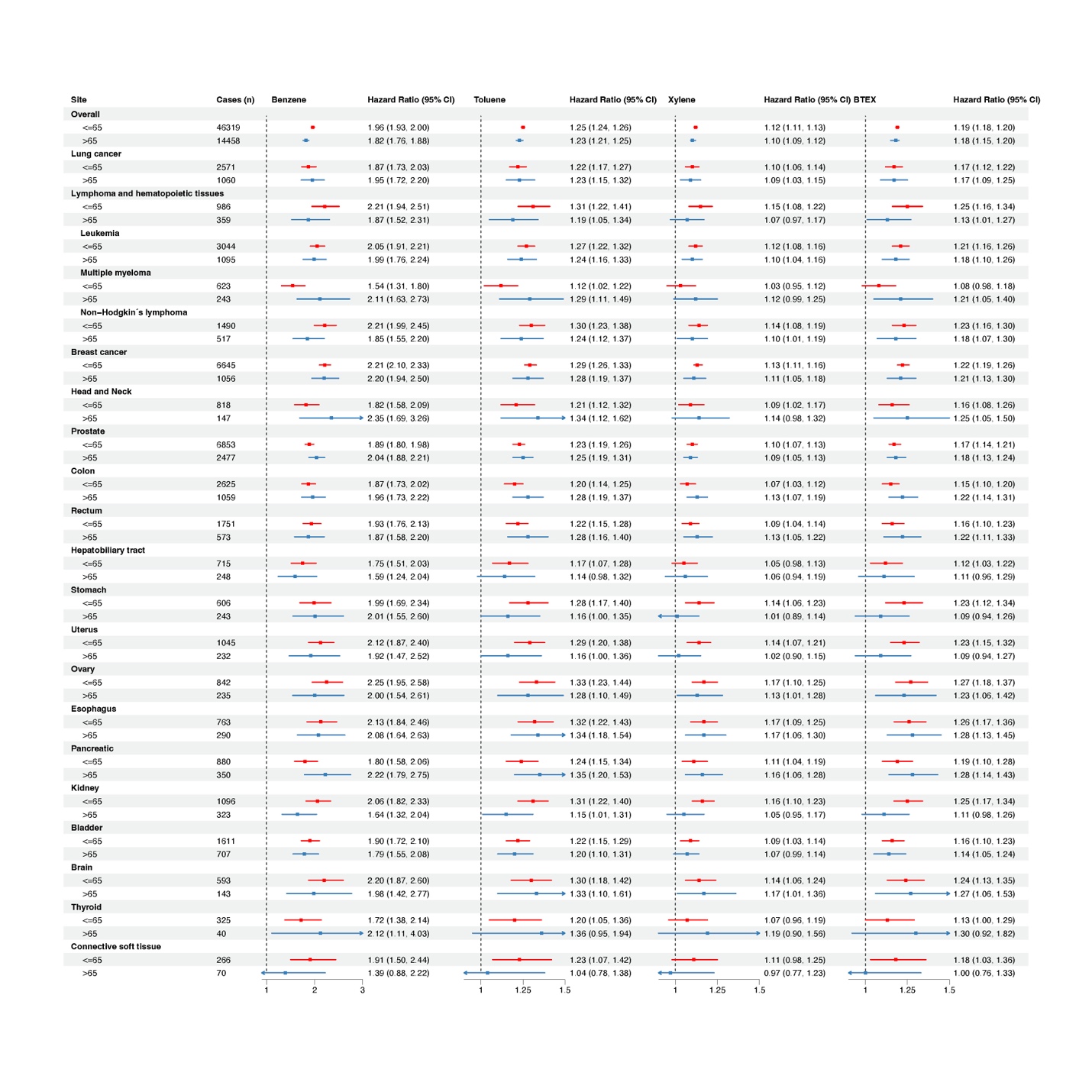


**Figure S4.** Associations of long-term exposure to benzene, toluene, and xylene with risk of overall and site-specific cancer stratified by age. Abbreviation: CI, confidence interval. Models were adjusted for sex, ethnicity, body mass index, drinking status, smoking status, physical activity, education qualification, household income, Townsend Deprivation Index, passive smoking exposure, solid-fuel usage, particulate matter with an aerodynamic diameter ≤ 2.5 µm (PM_2.5_) and nitrogen dioxide (NO_2_).


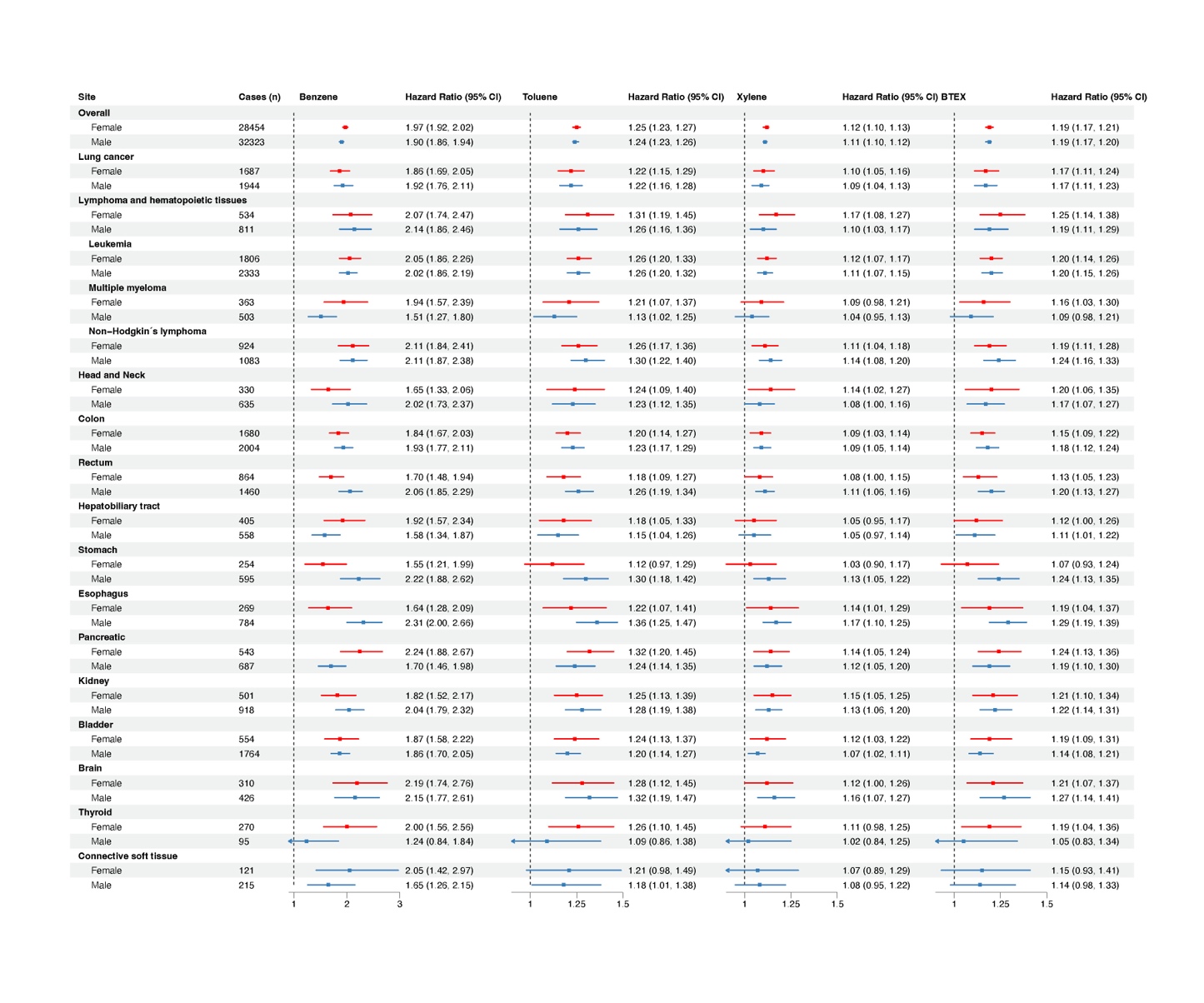


**Figure S5**. Associations of long-term exposure to benzene, toluene, and xylene with risk of overall and site-specific cancer stratified by sex. Abbreviation: CI, confidence interval. Models were adjusted for age, ethnicity, body mass index, drinking status, smoking status, physical activity, education qualification, household income, Townsend Deprivation Index, passive smoking exposure, solid-fuel usage, particulate matter with an aerodynamic diameter ≤ 2.5 µm (PM_2.5_) and nitrogen dioxide (NO_2_).


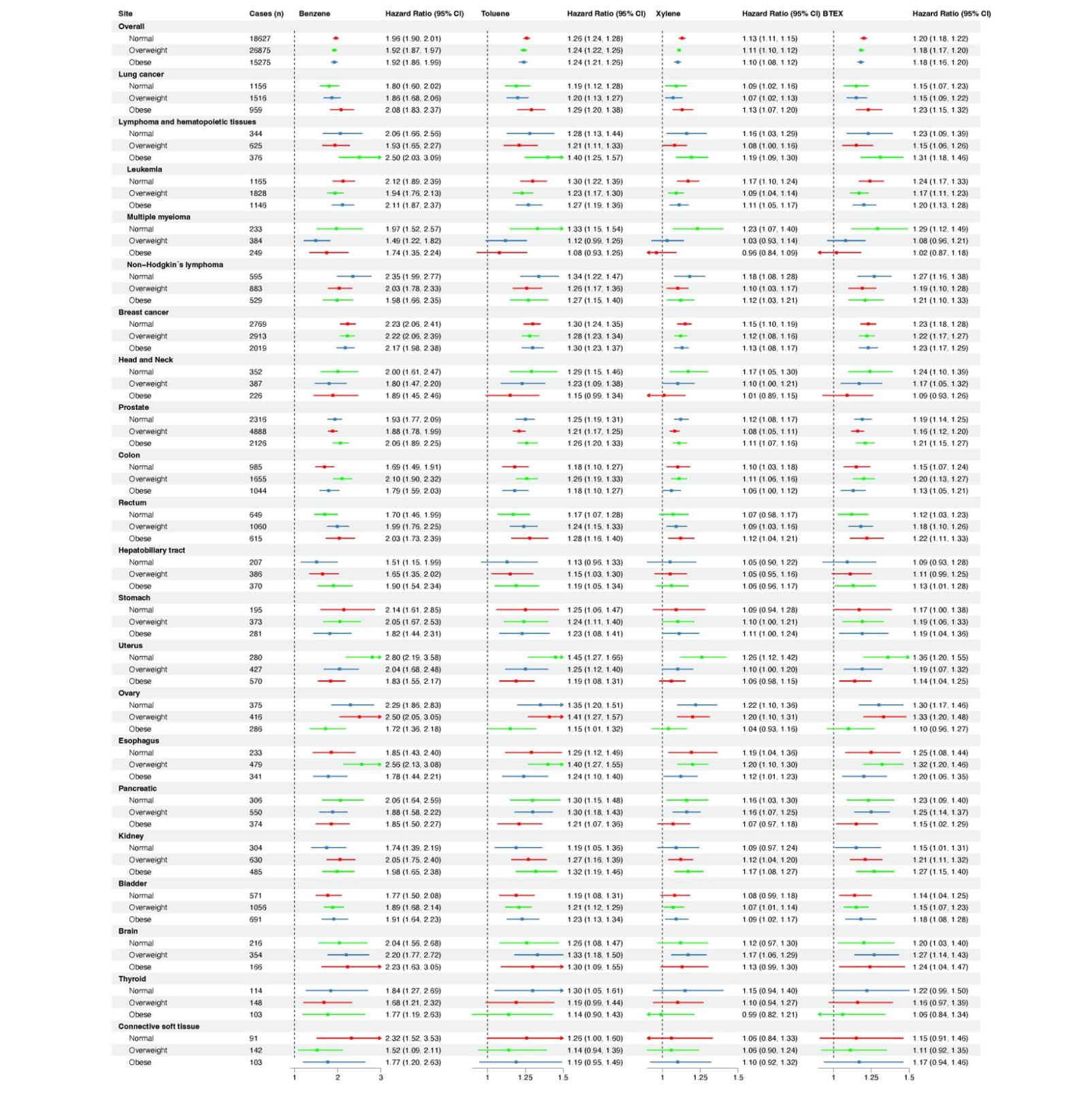


**Figure S6**. Associations of long-term exposure to benzene, toluene, and xylene with risk of overall and site-specific cancer stratified by body mass index. Models were adjusted for sex, age, ethnicity, drinking status, smoking status, physical activity, education qualification, household income, Townsend Deprivation Index, passive smoking exposure, solid-fuel usage, particulate matter with an aerodynamic diameter ≤ 2.5 µm (PM_2.5_) and nitrogen dioxide (NO_2_).


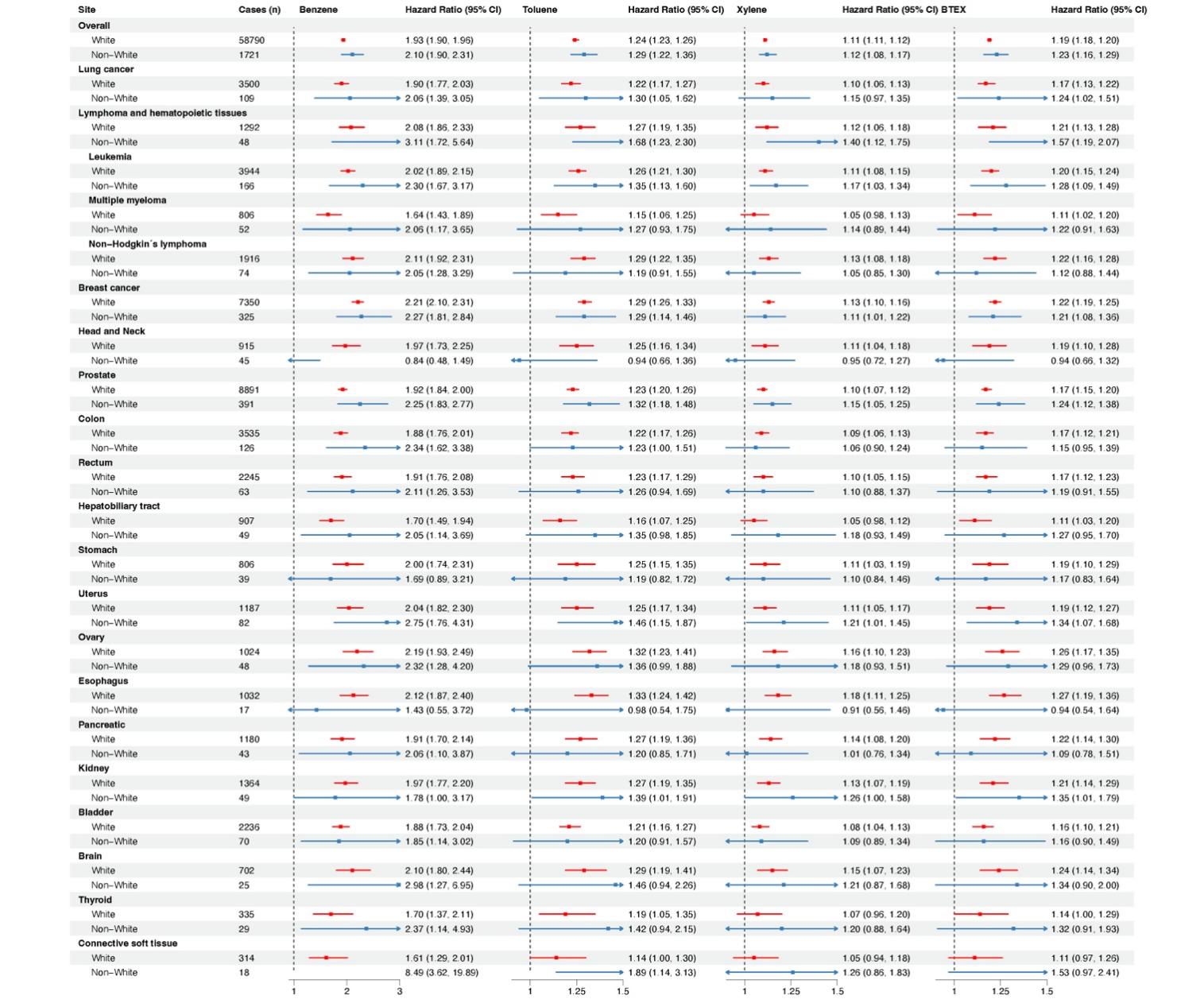


**Figure S7**. Associations of long-term exposure to benzene, toluene, and xylene with risk of overall and site-specific cancer stratified by ethnicity. Abbreviation: CI, confidence interval. Models were adjusted for sex, age, body mass index, drinking status, smoking status, physical activity, education qualification, household income, Townsend Deprivation Index, passive smoking exposure, solid-fuel usage, particulate matter with an aerodynamic diameter ≤ 2.5 µm (PM_2.5_) and nitrogen dioxide (NO_2_).


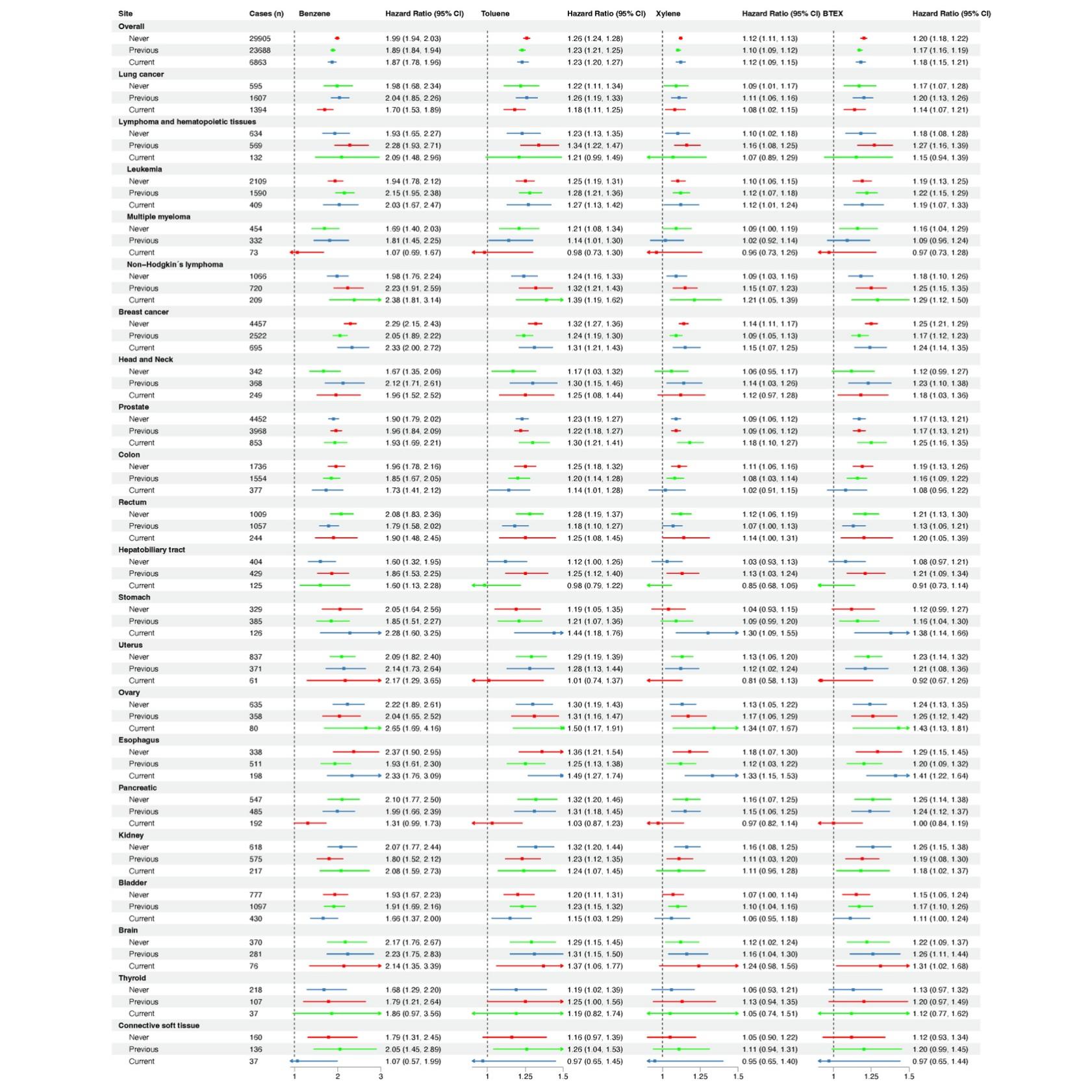


**Figure S8**. Associations of long-term exposure to benzene, toluene, and xylene with risk of overall and site-specific cancer stratified by smoking status. Abbreviation: CI, confidence interval. Models were adjusted for sex, age, body mass index, drinking status, ethnicity, physical activity, education qualification, household income, Townsend Deprivation Index, passive smoking exposure, solid-fuel usage, particulate matter with an aerodynamic diameter ≤ 2.5 µm (PM_2.5_) and nitrogen dioxide (NO_2_).


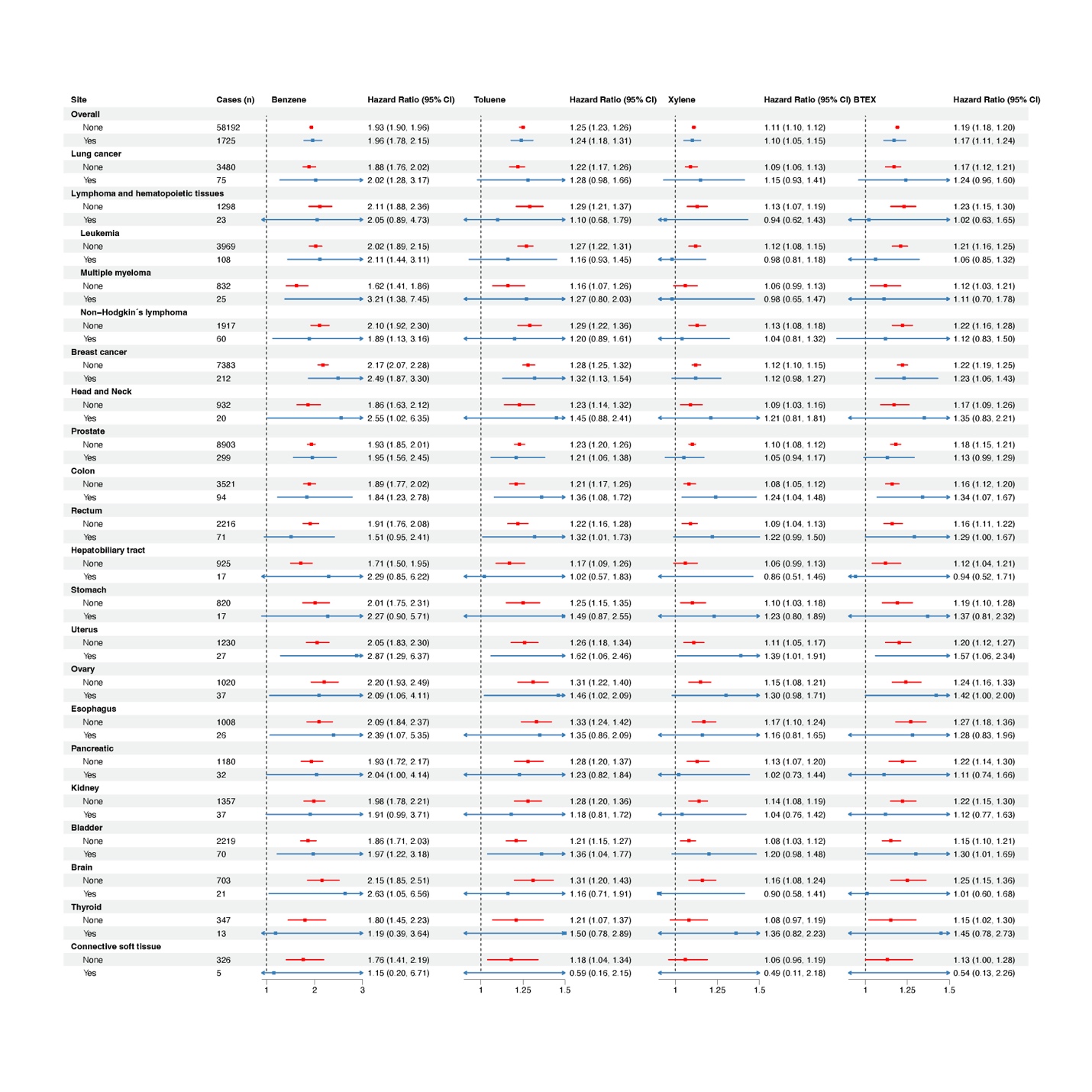


**Figure S9**. Associations of long-term exposure to benzene, toluene, and xylene with risk of overall and site-specific cancer stratified by solid-fuel usage. Abbreviation: CI, confidence interval. Models were adjusted for sex, age, body mass index, drinking status, ethnicity, smoking status, physical activity, education qualification, household income, Townsend Deprivation Index, passive smoking exposure, particulate matter with an aerodynamic diameter ≤ 2.5 µm (PM_2.5_) and nitrogen dioxide (NO_2_).

**
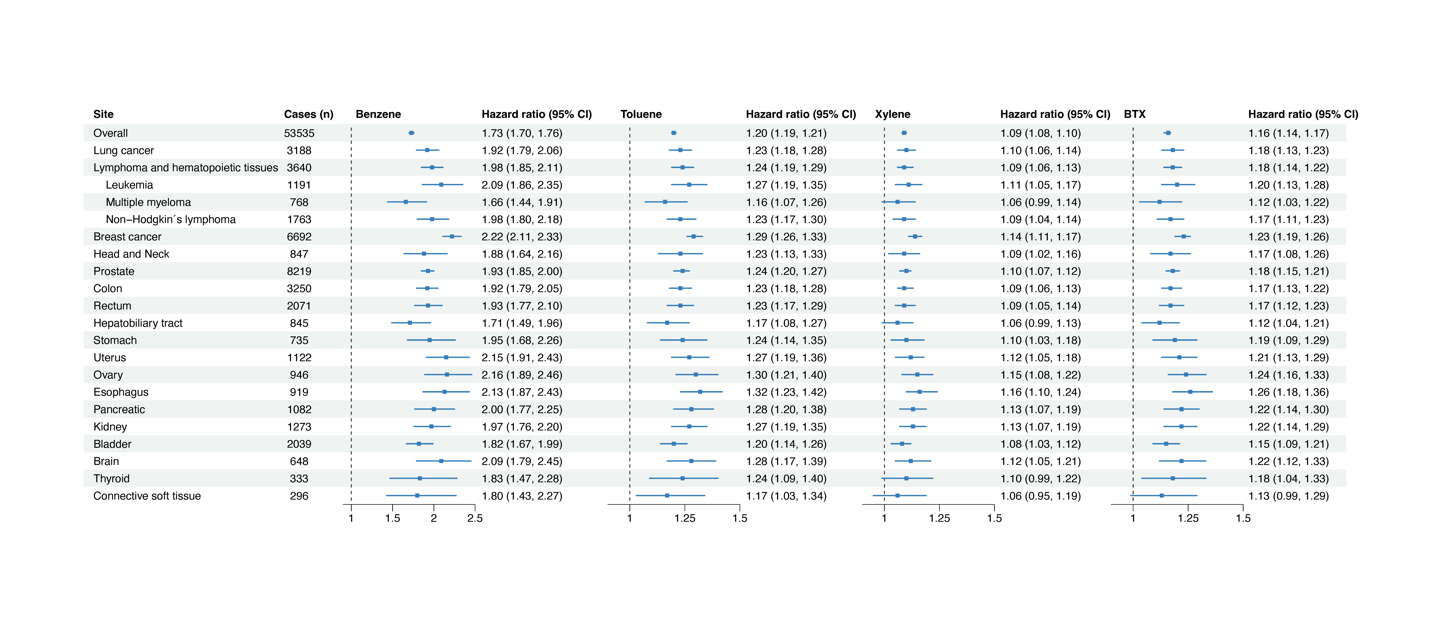
Figure S10.** Associations of long-term exposure to benzene, toluene, and xylene with risk of overall and site-specific cancer excluding cancer cases occurring over first 2 years of follow-up. Abbreviation: CI, confidence interval. Models were adjusted for age, sex, ethnicity, body mass index, drinking status, smoking status, physical activity, education qualification, household income, Townsend Deprivation Index, passive smoking exposure, solid-fuel usage, particulate matter with an aerodynamic diameter ≤ 2.5 µm (PM_2.5_) and nitrogen dioxide (NO_2_).

**
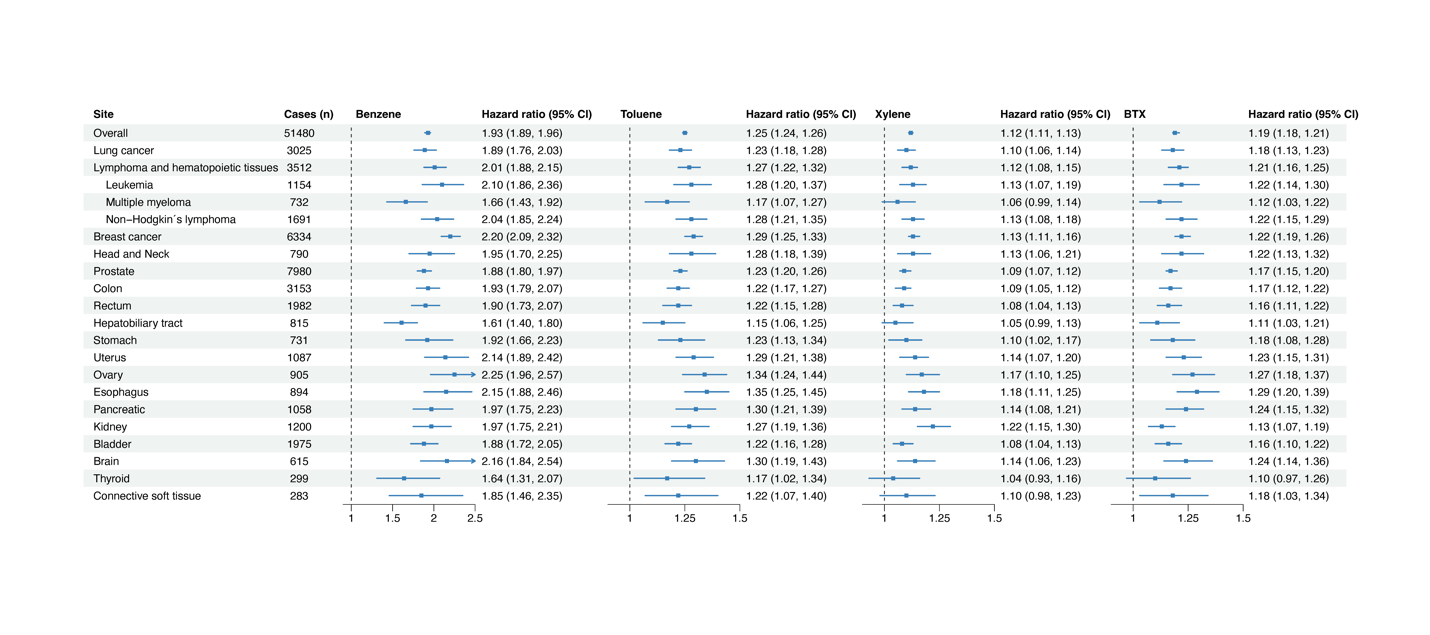
Figure S11.** Associations of long-term exposure to benzene, toluene, and xylene with risk of overall and site-specific cancer restricting to non-movers during the follow-up period. Abbreviation: CI, confidence interval. Models were adjusted for age, sex, ethnicity, body mass index, drinking status, smoking status, physical activity, education qualification, household income, Townsend Deprivation Index, passive smoking exposure, solid-fuel usage, particulate matter with an aerodynamic diameter ≤ 2.5 µm (PM_2.5_) and nitrogen dioxide (NO_2_).
